# Clinical importance of patient-reported outcome measures in severe asthma: results from U-BIOPRED

**DOI:** 10.1186/s12955-024-02321-3

**Published:** 2024-12-20

**Authors:** Roy Meys, Frits M.E. Franssen, Alex J. Van ‘t Hul, Per S. Bakke, Massimo Caruso, Barbro Dahlén, Stephen J. Fowler, Thomas Geiser, Peter H. Howarth, Ildikó Horváth, Norbert Krug, Annelie F. Behndig, Florian Singer, Jacek Musial, Dominick E. Shaw, Paolo Montuschi, Anke H. Maitland-van der Zee, Peter J. Sterk, Graham Roberts, Nazanin Z. Kermani, Raffaele A. Incalzi, Renaud Louis, Lars I. Andersson, Scott S. Wagers, Sven-Erik Dahlén, Kian Fan Chung, Ian M. Adcock, Martijn A. Spruit, on behalf of the U-BIOPRED Study Group

**Affiliations:** 1 Department of Research and Development, Hornerheide 1, 6085 NM, Ciro, Horn, The Netherlands; 2 NUTRIM School of Nutrition and Translational Research in Metabolism, Faculty of Health, Medicine and Life Sciences, Maastricht University, Maastricht, The Netherlands; 3 Department of Respiratory Medicine, Maastricht University Medical Centre (MUMC+), Maastricht, The Netherlands; 4 Department of Pulmonary Diseases, Radboud Institute for Health Sciences, Radboud University Medical Centre, Nijmegen, The Netherlands; 5 Department of Clinical Science, University of Bergen, Bergen, Norway; 6 Department of Biomedical and Biotechnological Sciences, University of Catania, Catania, Italy; 7 Lung/Allergy Clinic, Karolinska University Hospital Huddinge, Stockholm, Sweden; 8 Division of Infection, Immunity and Respiratory Medicine, School of Biological Sciences, Faculty of Biology, Medicine and Health, NIHR Biomedical Research Centre, University of Manchester and Manchester Academic Health Science Centre, Manchester University Hospitals NHS Foundation Trust, Manchester, UK; 9 Department of Pulmonary Medicine, University Hospital and University of Bern, Bern, Switzerland; 10 NIHR Southampton Biomedical Research Centre, Faculty of Medicine, University of Southampton, Southampton, UK; 11 Department of Pulmonology, Semmelweis University, Budapest, Hungary; 12 National Koranyi Institute for Pulmonology, Budapest, Hungary; 13 Fraunhofer Institute for Toxicology and Experimental Medicine Hannover, Hannover, Germany; 14 Department of Public Health and Clinical Medicine, Umeå University, Umeå, Sweden; 15 Paediatric Respiratory Medicine, Children’s University Hospital of Bern, University of Bern, Bern, Switzerland; 16 Division of Paediatric Pulmonology and Allergology, Department of Paediatrics and Adolescent Medicine, Medical University of Graz, Graz, Austria; 17 Department of Medicine, Jagiellonian University Medical College, Krakow, Poland; 18 Respiratory Research Unit, University of Nottingham, Nottingham, UK; 19 Catholic University of the Sacred Heart, Rome, Italy; 20 National Heart and Lung Institute, Imperial College, London, UK; 21 Amsterdam University Medical Center, University of Amsterdam, Amsterdam, The Netherlands; 22 The David Hide Asthma and Allergy Research Centre, St Mary’s Hospital, Newport, UK; 23 Università Campus Bio-Medico IT, Rome, Italy; 24 Department of Respiratory Medicine, GIGA I 3, CHU Sart-TilmanB35, University of Liege, Liege, Belgium; 25 Institute of Environmental Medicine, Karolinska Institutet, Stockholm, Sweden; 26 Centre for Allergy Research, Karolinska Institutet, Stockholm, Sweden; 27 BioSci Consulting, Maasmechelen, Belgium; 28 Department of Respiratory Medicine and Allergy, Karolinska University Hospital, Stockholm, Sweden; 29 Respiratory Department, Royal Brompton & Harefield Hospital, London, UK

**Keywords:** Obstructive pulmonary diseases, Patient outcome assessment, Health status

## Abstract

**Rationale:**

Knowledge about the clinical importance of patient-reported outcome measures (PROMs) in severe asthma is limited.

**Objectives:**

To assess whether and to what extent asthma exacerbations affect changes in PROMS over time and asthma-specific PROMs can predict exacerbations in adult patients with severe asthma in usual care.

**Methods:**

Data of 421 patients with severe asthma (62% female; mean age 51.9 ± 13.4 years; mean FEV_1_ 67.5 ± 21.3%pred) from the U-BIOPRED cohort were analyzed. The included PROMs were: Asthma Control Questionnaire (ACQ5); Asthma Quality of Life Questionnaire (AQLQ); Hospital Anxiety and Depression scale (HADS); Epworth Sleepiness Scale (ESS); Medication Adherence Report Scale (MARS); Sino-Nasal Outcomes Test (SNOT20). Participants were assessed at baseline and after 12–18 months of usual care.

**Results:**

PROMs showed very weak to weak correlations with clinical characteristics such as age, body mass index, FEV_1_, FeNO and eosinophilic cell count. Patients presenting no exacerbations during follow-up showed a statistically significant improvement in all PROMs (except for MARS), whereas individuals experiencing > 2 exacerbations showed a deterioration. Baseline ACQ5 was a predictor of exacerbations with an AUC of 0.590 (95%CI 0.514–0.666).

**Conclusions:**

The association of PROMs with clinical measures was poor in severe asthmatics. Moreover, PROMs were prone to changes in usual care, with exacerbations playing a key role. PROMs need to be systematically evaluated in severe asthma to improve clinical care based on specific patient’s needs.

**Supplementary Information:**

The online version contains supplementary material available at 10.1186/s12955-024-02321-3.

## Introduction

Severe asthma is a prevalent and heterogeneous condition that is defined by frequent asthma exacerbations and comorbidities, such as rhinosinusitis, allergies, obesity and psychopathologies [1]. In addition to its defining symptoms and impact on lung function, asthma may have extra-pulmonary manifestations, which also have a considerable impact on patient’s health and quality of life [2]. Initially, severe asthma treatment was focused on physiological indices of health, such as improving pulmonary function. However, the forced expiratory volume in the first second (FEV_1_) correlates weakly with patients’ quality of life [3]. Therefore, a true patient-centered approach needs to take the patient’s perspective into account, as some consequences of the disease can only be reported by patients [2]. Patient-reported outcome measures (PROMs) can be used to assess daily symptoms and understand the impact of severe asthma and the burden of its treatment [4].

PROMs show promise as being useful to support clinical decision-making [5]. However, asthma-related PROMs have mostly been evaluated in research contexts, for example with the aim of evaluating the effects of interventions on quality of life [6–8]. Knowledge about changes in PROMs in standard clinical asthma care, in which asthma exacerbations play a major role and outcomes cannot be directly attributed to specific experimental interventions, is scarce and mostly limited to respiratory-specific outcomes [9, 10]. It seems reasonable to hypothesize that asthma patients who experience exacerbations are prone to changes over time in both asthma-specific as well as more generic PROMs. Accordingly, the aims of the current study were to assess whether and to what extent: (1) asthma exacerbations affect changes in PROMS over time in usual asthma care and, (2) asthma-specific PROMs can predict asthma exacerbations in adult patients with severe asthma.

## Methods

This is an analysis of the data from the Unbiased Biomarkers for the Prediction of Respiratory Disease Outcomes project (U-BIOPRED; NCT01982162, registered October 30, 2013), a multicenter prospective longitudinal cohort study in which 610 adults (18 + years) from 16 clinical institutions across Europe were recruited between May 2011 and April 2013 [11]. The current study included the 421 adult patients with *severe asthma* from the cohort, which was defined as having uncontrolled symptoms and/or frequent (≥ 2 per year) exacerbations despite high intensity asthma treatment (≥ 1000 µg/day fluticasone equivalent and/or daily oral corticosteroids combined with long-acting β2 agonists or any other controller medication) [12]. Asthma diagnosis was confirmed by a history of wheeze (either spontaneously or on exertion), as well as variable airflow limitation by one of the following: airflow reversibility (increase in forced expiratory volume in 1s (FEV_1_) > 12% predicted and 200 mL following inhalation of 400 µg salbutamol), airway hyperresponsiveness (methacholine provocative concentration causing a 20% fall in FEV_1_ < 8 mg·mL − 1, a diurnal peak expiratory flow amplitude > 8% of mean), or a decrease in FEV_1_ of 12% predicted and 200 mL within 4 weeks after tapering maintenance treatment [12]. Participants with severe asthma were reviewed at baseline and 12–18 months after enrolment. The medical ethics committee of each participating center approved this study and all patients gave written informed consent. Baseline data, details of the participating centers and standard operating procedures of the U-BIOPRED project have been published [11]. Moreover, studies focusing on self-reported medication adherence, using the Medication Adherence Report Scale (MARS) [13], and treatable traits in the adult U-BIOPRED cohort [14] have been performed.

### PROMs

Asthma symptom control was measured with the Asthma Control Questionnaire (ACQ5), a self-administered 5-item questionnaire with scores ranging from 0 (totally controlled) to 6 (severely uncontrolled) points [15]. A cut-off score of > 1.5 points was used to identify patients with uncontrolled asthma [16]. To evaluate quality of life, the Asthma Quality of Life Questionnaire (AQLQ) was administered [17]. The AQLQ consists of 32 questions (7-point Likert scale), covering 4 domains (symptoms, activity limitation, emotional function and environmental exposure), with higher scores indicating better quality of life. An AQLQ score < 4.7 points was used a cut-off value for impaired quality of life [18].

Mood status was measured with the 14-item Hospital Anxiety and Depression scale (HADS; range 0–21 points per domain) [19]. Cut-off scores of ≥ 8 points were used to identify patients with elevated levels of anxiety or depression [20]. Sleep propensity in daily life or ‘daytime sleepiness’ was determined with the Epworth Sleepiness Scale (ESS) [21], a self-administered questionnaire containing 8 questions with a 4-point scale (0–3). A higher score represents more daytime sleepiness and a cut-off of ≥ 11 points was used to indicate a high risk on excessive daytime sleepiness (ESS) [21]. The MARS is a 5-item measure of self-reported adherence, in which a cut-off of < 23 points was applied to indicate poor adherence [13, 22]. Upper airway symptoms were assessed with the Sino-Nasal Outcomes Test (SNOT20) [23], a 20-question survey in which a higher score indicates greater impairment. A score of ≥ 2 points was considered abnormal [24].

### Other assessments

Demographics, body mass index (BMI, body weight in kilograms divided by the height in squared meters), smoking history and medication use were recorded. Both at the baseline and the follow up visit, participants were asked to report the number of exacerbations that needed systemic corticosteroid therapy in the previous 12 months. In addition, the presence of the following comorbidities were captured: allergic rhinitis, non-allergic rhinitis, nasal polyps, laryngeal dysfunction, chronic sinusitis, hay fever, emphysema, psychopathologies, atopic dermatitis, obesity (BMI ≥ 30), gastroesophageal reflux disease (GERD), congestive heart failure, coronary heart disease, hypertension, diabetes, osteoporosis. To measure pulmonary function, participants underwent fractional exhaled nitric oxide (FeNO) testing at 50 mL/s and spirometric measurements (FEV_1_) [25, 26]. Allergic sensitization was obtained by measuring specific immunoglobulin (IgE) to six common aeroallergens or skin prick testing [27]. Blood samples were obtained for measurement of hematological indices, such as eosinophilic and neutrophilic cell count as well as for total IgE. Procedures are detailed further in previous work [11].

### Statistics

The data of the current study was downloaded in February 2022 from tranSMART, an open-source knowledge management platform, after which analyses were performed using IBM SPSS Statistics 25.0 (SPSS Inc., Chicago, USA) and GraphPad Prism 9.0 (GraphPad Software Inc., California, USA). Results are presented as mean and standard deviation (SD), median and interquartile range (IQR), and/or frequencies, as appropriate. Continuous variables were tested for normality. The interrelationship between PROMs and the relationship between PROMs and clinical characteristics were analyzed using Spearman’s correlations. The strength of correlations has been classified according to British Medical Journal (BMJ) guidelines, which classify significant correlation coefficients of 0–0.19 as very weak, 0.2–0.39 as weak, 0.4–0.59 as moderate, 0.6–0.79 as strong, and 0.8–1 as very strong [28]. Differences between the baseline and longitudinal assessment were analyzed using paired T-tests or Wilcoxon signed rank test, as appropriate. To assess the effect of asthma exacerbations, differences in PROM scores at baseline and changes in PROMs were compared after stratification into three groups based on the number of exacerbations in the preceding 12 months (0 exacerbations; 1–2 exacerbations; >2 exacerbations) recorded at the longitudinal follow-up. Between-group differences were analyzed using Analysis of Variance (ANOVA) with Bonferroni post-hoc correction. Finally, to assess the diagnostic value of asthma-specific PROMs in terms of predicting an asthma exacerbation, the AUC (Area Under The Curve) ROC (Receiver Operating Characteristics) curve was calculated. The ROC analysis results were interpreted as follows: AUC < 0.70, low diagnostic accuracy; AUC 0.70–0.90, moderate diagnostic accuracy; and AUC ≥ 0.90, high diagnostic accuracy [29]. A priori, the level of significance was set at ≤ 0.05.

## Results

Patient characteristics and baseline PROM scores of the 421 included patients are presented in Table 1. The majority of patients were female (62%), with a mean age of 51.9 ± 13.4 years. One-hundred-and-sixty-four patients (39%) were obese and the mean FEV_1_ was 67.5 ± 21.3% of predicted. The median number of experienced asthma exacerbations in the 12 preceding months was 2 (IQR 1–3), whereas the median number of comorbidities at baseline was 4 (2–5). The mean ACQ5 score at baseline was 2.28 ± 1.17 points and 74% of the patients were classified as having uncontrolled asthma. The mean baseline AQLQ score was 4.45 ± 1.21 points and in 57% of the patients an impaired asthma-related quality of life was observed. Mean HADS-A and HADS-D scores were 7.1 ± 4.5 points and 5.6 ± 4.6 points, with 44% and 30% of patients presenting increased levels of anxiety and depression (values above the ≥ 8 points cut-off), respectively.

**Table 1:** Baseline characteristics and PROM scores

Clinical characteristics	Age, years	51.9 ± 13.4
	Age at diagnosis, years	26.0 (9.0–42.0)
	Females, n (%)	261 (62.0)
	BMI, kg/m^2^	29.2 ± 6.3
	BMI > 30 kg/m^2^, n (%)	164 (39.0)
	Smoking: current/former/never, n (%)	42 (10) /115 (27) /264 (63)
	Serum IgE, IU/mL	122 (51–350)
	Atopy test positive, n (%)	275 (64.3)
	FEV_1_, %predicted	67.5 ± 21.3
	FVC, %predicted	87.9 ± 19.3
	FEV_1_/FVC ratio	0.63 ± 0.14
	FeNO, ppb	26 (15–47)
	Blood neutrophils, cells/µL	4810 (3700–6778)
	Blood eosinophils, cells/µL	200 (100–400)
	Asthma exacerbations < 12 months, n	2 (1–3)
	0 exacerbations, n (%)	79 (18.8)
	1–2 exacerbations, n (%)	177 (42.1)
	≥ 3 exacerbations, n (%)	164 (39.0)
	Comorbidities, n	4 (2–5)
	≥ 2 comorbidities, n (%)	362 (86.0)
PROMs	ACQ5, points	2.28 ± 1.17
	ACQ5 > 1.5 points, n (%)	285/388 (73.5)
	AQLQ, points	4.45 ± 1.21
	AQLQ < 4.7 points, n (%)	237/414 (57.2)
	ESS, points	7.52 ± 4.47
	ESS ≥ 11 points, n (%)	99/386 (25.6)
	HADS-A, points	7.14 ± 4.52
	HADS-A ≥ 8 points, n (%)	173/391 (44.2)
	HADS-D, points	5.63 ± 4.56
	HADS-D ≥ 8 points, n (%)	118/391 (30.2)
	MARS, points	22.37 ± 2.47
	MARS < 23 points, n (%)	158/404 (39.1)
	SNOT20, points	1.59 ± 0.88
	SNOT20 > 2 points, n (%)	102/347 (29.4)

Summary variables are presented as n (%) for discrete variables, mean ± standard deviation for quantitative variables or median (Interquartile range) for skewed variables

*Abbreviations*: PROMs, patient-reported outcomes; BMI, body mass index; kg/m^2^, kilogram per square meter; IgE, Immunoglobuline E; IU/mL, international units per milliliter; FEV_1_, forced expiratory volume in the first second; FVC, forced vital capacity; FeNO, fractional exhaled nitric oxide; ppb, parts per billion; cells/µL, cells per microliter; ACQ5, Asthma Control Questionnaire; AQLQ, Asthma Quality of Life Questionnaire; ESS, Epworth Sleepiness Scale; HADS-A, Hospital Anxiety and Depression Scale, Anxiety subscale; HADS-D, Hospital Anxiety and Depression Scale, Depression subscale; MARS, Medication Adherence Report Scale; SNOT20, Sino-Nasal Outcomes Test

### Correlations PROMs and clinical characteristics

All PROMs showed very weak or non-significant correlations with clinical characteristics (age, BMI, FEV_1_%pred, FeNO, eosinophilic and neutrophilic cell count and number of exacerbations in the preceding 12 months) at baseline (Table 2). Weak correlations were only found between asthma control (ACQ5) and FEV_1_%pred or the number of exacerbations in the past 12 months, and between quality of life (AQLQ) and FEV_1_%pred (ρ: 0.23–0.25; all *p* < 0.001). The interrelationship of the PROMs in shown in Table E1. In brief, the correlation between the asthma control (ACQ5) and quality of life (AQLQ) and the correlation between depression and anxiety (HADS-D and HADS-A) were the strongest (ρ: -0.77 and 0.75, respectively; *p* < 0.001).

**Table 2:**
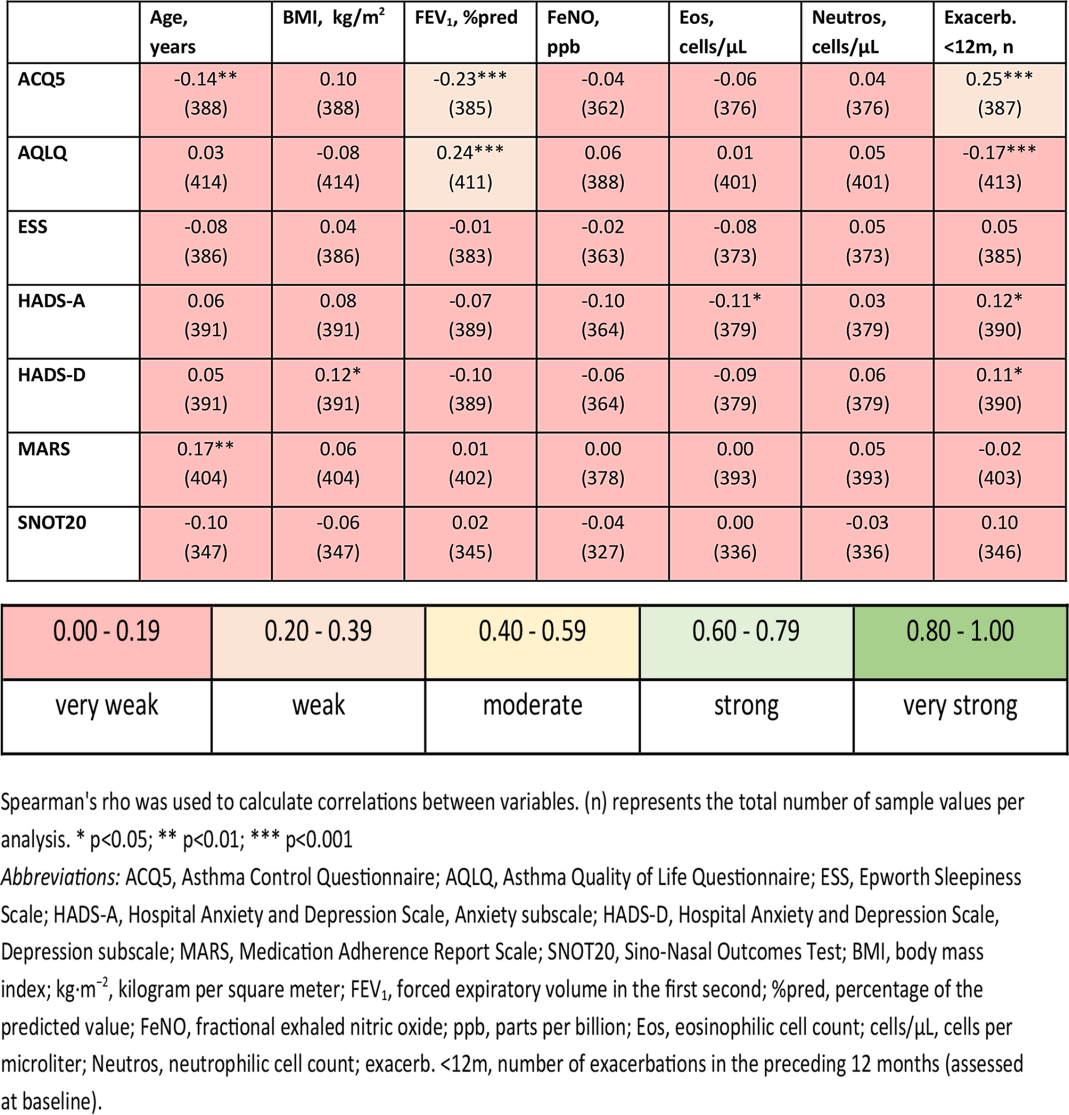
Correlations PROMs and clinical characteristics

### Changes in PROMs

The median time between the baseline and follow-up visit was 444 (IQR 400–514) days. Patients who did not consent to participate in the longitudinal assessment (*n* = 104) and patients who attended the follow-up visit less than 365 days after the baseline visit (*n* = 17) were excluded, resulting in 300 patients (71.3%) for the longitudinal analyses. Patients who attended the longitudinal visit (*n* = 300) and excluded patients (*n* = 121) were comparable regarding baseline characteristics (Table E2).

### Changes in PROMs after stratification for exacerbations

After stratification for the number of asthma exacerbations during follow-up (0 *versus* 1–2 *versus* > 2), multiple differences were observed at follow-up (Table 3). The group of patients who did not experience any asthma exacerbation during follow-up showed a statistically significant improvement in all PROMs (except for MARS score), while FEV_1_, FeNO and blood eosinophils did not change significantly over this time-frame. The groups of individuals experiencing > 2 asthma exacerbations during the follow-up period deteriorated in terms of PROM scores, even though this group already presented the worse PROM scores at baseline and showed no changes in any of the clinical characteristics (Table 3).

**Table 3:** Differences in PROM scores at baseline and changes in PROMs after stratification based on the number of exacerbations in the preceding 12 months (recorded at the longitudinal follow-up)

		0 exacerbations (*n* = 84)	1–2 exacerbations (*n* = 100)	> 2 exacerbations (*n* = 116)	Between group difference *p*-value
Clinical characteristics	Age, baseline, years	52.6 ± 14.7	54.4 ± 12.6	51.0 ± 12.5	0.178
	Age at diagnosis, years	29 (11–44)	26 (7–44)	25 (7–37)	0.574
	Female sex, n (%)	43 (51.2)	60 (60.0)	76 (65.5)	0.125
	Follow-up, days	455 (393–520)	446 (404–510)	441 (402–511)	0.600
	BMI, baseline, kg/m^2^	28.3 ± 5.0	29.2 ± 6.3	29.4 ± 6.3	0.429
	Comorbidities (baseline), n	4 (2–5)	4 (2–5)	4 (2–6)	0.324
	≥ 2 comorbidities	76 (90.5)	84 (84.0)	98 (84.5)	0.377
	Exacerbations < 12 months (baseline), n	1 (0–2)	2 (1–3)	3 (2–4)^#&^	< 0.001
	Blood eosinophils, cells/µL	220 (100–460)	230 (120–500)	200 (100–400)	0.449
	ΔBlood eosinophils, cells/µL	-0.3 (-100–64)	-1.18 (-170–100)	0 (-99–152)	0.678
	FEV1, %pred (baseline)	64.1 ± 18.8	67.5 ± 21.6	66.5 ± 21.9	0.526
	ΔFEV1, %pred	0.7 ± 12.7	1.8 ± 14.3	-0.9 ± 14.8	0.386
	FeNO, ppb	27 (15–39)	29 (16–58)	24 (15–45)	0.460
	ΔFeNO, ppb	-1 (-15–8)	2 (-13–9)	0 (-9–6)	0.540
	Tapering of ICS, n (%)	8 (9.5)	12 (12.1)	20 (17.2)	0.258
	Tapering of OCS, n (%)	3 (3.6)	12 (12.0)	20 (17.2)	< 0.05
PROMs	ACQ5, baseline score, points	2.03 ± 1.17	2.12 ± 1.18	2.62 ± 1.13^#&^	< 0.001
	ΔACQ5, points	-0.44 ± 1.08*	-0.09 ± 1.03	0.28 ± 1.02^#&^	< 0.001
	ΔACQ5 ≤ − 0.5, n (%)	33 (39.3)	30 (30.0)	18 (15.5)	< 0.001
	AQLQ, baseline score, points	4.60 ± 1.26	4.65 ± 1.23	4.21 ± 1.08	< 0.05
	ΔAQLQ, points	0.30 ± 0.96*	0.18 ± 0.71*	-0.13 ± 0.74^#&^	< 0.01
	Δ AQLQ ≤ + 0.5, n (%)	34 (40.5)	26 (26.0)	14 (12.1)	< 0.001
	ESS, baseline score, points	8.00 ± 4.33	7.92 ± 4.52	8.13 ± 4.56	0.949
	ΔESS, points	-1.03 ± 4.29*	0.09 ± 3.33	0.49 ± 3.15^#^	< 0.05
	HADS-A, baseline score, points	6.99 ± 4.53	6.32 ± 4.42	7.74 ± 4.63	0.113
	ΔHADS-A, points	-0.89 ± 3.52*	0.27 ± 3.06	0.47 ± 3.35^#^	< 0.05
	HADS-D, baseline score, points	5.81 ± 4.27	5.23 ± 4.62	5.90 ± 4.60	0.568
	ΔHADS-D, points	-1.01 ± 3.22*	-0.14 ± 3.17	0.12 ± 3.21	0.066
	MARS, baseline score, points	22.07 ± 2.75	22.79 ± 2.10	22.00 ± 2.41	< 0.05
	ΔMARS, points	-0.27 ± 3.01	0.16 ± 1.90	0.10 ± 1.95	0.410
	SNOT20, baseline score, points	1.55 ± 0.82	1.46 ± 0.86	1.86 ± 0.95^&^	< 0.05
	ΔSNOT20, points	-0.21 ± 0.76*	0.04 ± 0.58	0.10 ± 0.77^#^	< 0.05

Summary variables are presented as n (%) for discrete variables, mean ± standard deviation for quantitative variables or median (Interquartile range) for skewed variables. * *p* < 0.05 pre vs. post (paired samples T-test); # *p* < 0.05 0 exacerbations vs. > 2 exacerbations group; & *p* < 0.05 1–2 exacerbations vs. > 2 exacerbations group

*Abbreviations*: PROMs, patient-reported outcomes; BMI, body mass index; kg/m^2^, kilogram per square meter; cells/µL, cells per microliter; FEV_1_, forced expiratory volume in the first second; %pred, percentage of the predicted value; FeNO, fractional exhaled nitric oxide; ppb, parts per billion; ICS, inhaled corticosteroids; OCS, oral corticosteroids; ACQ5, Asthma Control Questionnaire; AQLQ, Asthma Quality of Life Questionnaire; ESS, Epworth Sleepiness Scale; HADS-A, Hospital Anxiety and Depression Scale, Anxiety subscale; HADS-D, Hospital Anxiety and Depression Scale, Depression subscale; MARS, Medication Adherence Report Scale; SNOT20, Sino-Nasal Outcomes Test

### Prediction of asthma exacerbations using asthma-specific PROMs

ROC curve analysis revealed that baseline asthma control (ACQ5) was a significant discriminant factor in predicting at least one asthma exacerbation between baseline and the follow-up visit (Figure E1) with an AUC of 0.590 (95%CI 0.514–0.666), indicating low diagnostic accuracy. The ACQ5 cut-off value of > 1.5 points [16] represented 78.8% sensitivity and 62.8% specificity. The number of asthma exacerbations in the 12 months preceding the baseline assessment was the strongest predictor for an asthma exacerbation during follow-up, with an AUC 0.684 (95%CI 0.615–0.753; *p* < 0.001). Baseline quality of life (AQLQ score), FEV1%pred, age and BMI were no significant predictive factors regarding asthma exacerbations during the follow-up period (all *p* > 0.05; Figure E1).

## Discussion

This study demonstrates that all PROM scores improved significantly over time in patients with severe asthma who did not experience an asthma exacerbation in the 12 months preceding the follow-up measurement, while no changes were observed in clinical characteristics such as FEV_1_, FeNO or blood eosinophils. Contrastingly, patients who experienced more than two asthma exacerbations showed a deterioration in PROM scores, despite the fact that baseline scores were already significantly worse in these patients at baseline compared to those with fewer exacerbations during follow up. Furthermore, PROMs, which are generally impaired in patients with severe asthma, show a very weak or non-significant association with clinical characteristics, which highlights the importance of considering patient-reported outcomes to better understand the true impact of the disease on patients’ lives, and in turn, as one of the key outcomes. This well established decoupling between biological and patient-related measures in obstructive pulmonary disease [30, 31] testifies to the fact that the transition from the biological to the clinical/health status dimension is complex (i.e. explaining the mediating mechanisms) and should be considered when designing future trials on asthma, mainly for the selection of outcome variables.

The aim of the U-BIOPRED project was to comprehensively assess the impact of asthma on all domains relevant to patients in addition to measuring traditional and relatively new biomedical markers [11]. Therefore, multiple asthma-specific as well as more generic instruments such as the ESS (daytime sleepiness) and the HADS (anxiety and depression) were included. The most convincing interrelationship between PROMs was found between quality of life and asthma control, which has been shown before and seems to be the result of the fact that both questionnaires (AQLQ and ACQ5) are measuring strongly related concepts related to symptoms and impacts directly of asthma [3]. The weak-to-moderate interrelationship between the other PROMs in the current study clearly indicates that these questionnaires are not interchangeable, as they assess different aspects of the disease and therefore provide information on different dimensions or attributes other than asthma. However, this also raises uncertainty about which PRO data to use and underlines the fact that there is no “one-size-fits-all” approach for integrating PROs into clinical practice. In light of this, the PROTEUS- Consortium (Patient-Reported Outcomes Tools: Engaging Users & Stakeholders) was formed to specifically address the use of PROs in clinical practice [32]. The PROTEUS-Practice Guide identifies general barriers for using PROs in clinical care and highlights potential solutions to help overcome these challenges [32]. Despite substantial improvements in this area in recent years, PROMs remain rarely integrated in general asthma care, with specialistic centers being the only exempt. By incorporating PROMs, these centers are able to tailor patient treatment plans to individual patient needs, monitor disease progression and assess the impact of interventions [33, 34]. For instance, patients enrolled to the UK Severe Asthma Registry (UKSAR) after referral to Specialist UK Severe Asthma Centres are regularly assessed using PROMs to evaluate the impact of biologic therapies on patient-reported asthma control and quality of life [35]. The current study provides important insights in the impact of severe asthma as seen from the patient’s perspective, which shows a tendency to significantly change over a relatively short period of time, supporting the growing evidence to collect PROMs on a routinely basis [1, 34, 36], thereby enabling better and more patient-centered care.

The fact that baseline PROMs in the current cohort were already impaired in a considerable number of patients, which has not resulted in specific non-pharmacological treatments during follow-up, confirms the lack of clear guidance regarding the use of PROMs in clinical practice. In fact, poor self-reported adherence to medication was measured in 40% of the patients at baseline using the MARS questionnaire. However, no significant improvements in adherence were observed during follow-up across all three exacerbation groups. This underlines the fact that low adherence, whether measured directly or self-reported, is a common problem in severe asthma [13]. By combining several strategies such as patient education and fixed-dose regimens, healthcare providers are able to help patients with asthma improve their medication adherence, leading to better asthma control and overall health outcomes [37].

Prior to enrolment in the U-BIOPRED study, participants with severe asthma were required to have been under follow-up by a respiratory physician for at least 6 months, guided by assessments to optimize both asthma control and medication adherence [11]. Furthermore, patients in the included cohort were, on average, diagnosed with asthma for more than 25 years. Nevertheless, the current study was able to show that, when taking into account the prospective number of asthma exacerbations, asthma control and asthma-related quality of life were prone to clinically significant changes over the course of a little more than one year. This is highly relevant to the use of PROMs in clinical settings, as experiencing an asthma exacerbation during follow up seemed to affect almost every PROM in a negative way, whereas the group of patients who did not experience an exacerbation showed significant improvements over time in every PROM. These results underline the importance of “zero tolerance for asthma exacerbations”, as advocated by the Lancet Asthma Commission in 2017 [2], who urged the need to identify and advertise high-risk periods and to provide targeted and effective patient advice, seeking value in the development of a risk score [38], which could be incorporated into an annual examination, and might aid the primary prevention of asthma exacerbations.

Based on the current study it can be concluded that the accuracy of asthma-specific PROMs and clinical variables such as age and FEV_1_ in terms of predicting an asthma exacerbation is low. Only the amount of previous asthma exacerbations tended to be able to predict a future exacerbation, which is in line with a large population-based study [39]. In fact, it seems that in the general population not only those with more severe disease or higher levels of treatment present with asthma exacerbations, suggesting risk may reflect individual susceptibility, rather than simply being associated with severe asthma [40].

To date, little is known about the possibilities of improving PROMs in patients with severe asthma. Existing knowledge is mostly based on robust literature regarding indications and components of pulmonary rehabilitation (PR) in patients affected by COPD [41]. PR has been shown to be a successful intervention for these patients with clinically meaningful improvements in terms of symptoms, depression and health-related quality of life, irrespective of pulmonary function [42]. Although several studies also suggest an important role of non-pharmacological treatment in addition to pharmacological therapy in patients with asthma [43–45], future research is needed to improve clinical care based on specific patient’s needs while integrating the inherent information of PROMs into a comprehensive view of the patient.

To the best of our knowledge, the current study is the first to assess the course of multiple asthma-specific as well as more generic PROMs over time in patients with severe asthma, who are being treated based on routine asthma care, without receiving any experimental type of intervention. Another strength of the current study assessing PROMs is the fact that patients were actively involved in the development of the U-BIOPRED research protocol, as patient and public involvement (PPI) is becoming increasingly important when conducting research. Nevertheless, some limitations need to be acknowledged. Data collection was limited to only two time points. This limits the ability to identify fluctuations in symptom presentation and other contributors to changes in PROMs, with no direct linkage between the timing of exacerbations and the time of assessment. Future research should focus on measuring intermediate time-points with possibly, a longer follow-up period. The current study sample consisted of young adults on average and, thus, a different PROMs-asthma severity relationship might characterize subjects with late onset asthma, who also have a different pattern of comorbidity. Since comorbidities can exacerbate asthma symptoms and complicate its management, leading to worse health outcomes and lower quality of life for patients, treating comorbidities in patients with severe asthma is likely to improve PROMs for both asthma and the comorbid conditions. Lastly, severe asthma is increasingly recognized to be different from mild-to-moderate asthma, in particular regarding the impact of hospitalizations and the frequent use of OCS [1]. The PROMs used in the current study have not been developed specifically for severe asthma and have not been assessed regarding their discriminative, classificatory and prognostic properties in this population. These PROMs might therefore fail to assess factors that are unique to severe asthma. Nonetheless, all of the included PROMs have been shown to differ between patients with severe asthma and patients with mild/moderate asthma, with all outcomes being far worse in the former group [11]. Future methodological studies assessing the clinimetric properties of both existing and new PROMS such as the Severe Asthma Questionnaire (SAQ) [46] are needed to select the ones most suitable to patients with severe asthma.

In conclusion, PROMs are significantly impaired in patients with severe asthma, with asthma-specific outcomes (e.g. asthma control and asthma-related quality of life) being impacted the most. Asthma exacerbations seem to be the main drivers of changes (either an improvement or a deterioration) in ACQ5 and AQLQ scores during follow up while receiving standard care. The very weak or non-existent correlation with clinical measures in this sample, emphasizes the need to systematically evaluate PROMs in the clinical care of patients with severe asthma and to take into account the perception of disease impact. Once more, it has been proven that patients with similar clinical characteristics can present different responses to the impact of symptoms on their lives, supporting the evolution to more personalized, patient-focused disease management. Profound phenotyping using high-dimensional molecular biomarkers is necessary to identify associations that are not displayed in this first PROMs analysis in severe asthmatics.

## Electronic supplementary material

Below is the link to the electronic supplementary material.

Supplementary Material 1

## Data Availability

The datasets generated during and/or analyzed during the current study are available on reasonable request.
