# Supplementary material for: Clinical importance of patient-reported outcome measures in severe asthma: results from U-BIOPRED
Source: Health Qual Life Outcomes. 2024 Dec 20;22:109. doi: 10.1186/s12955-024-02321-3 (PMC11662558; doi:10.1186/s12955-024-02321-3)
Supplement: Supplementary file 1 — Supplementary Material 1 [file 12955_2024_2321_MOESM1_ESM.docx]

***Online Data Supplement***

**Clinical importance of patient-reported outcome measures in severe asthma: results from U-BIOPRED**

Roy Meys, Frits M.E. Franssen, Alex J. van ‘t Hul, Per S. Bakke, Massimo Caruso, Barbro Dahlén, Stephen J. Fowler, Thomas Geiser, Peter H. Howarth, Ildikó Horváth, Norbert Krug, Annelie F. Behndig, Florian Singer, Jacek Musial, Dominick E. Shaw, Paolo Montuschi, Anke-Hilse Maitland-van der Zee, Peter J. Sterk, Graham Roberts, Nazanin Z. Kermani, Raffaele A. Incalzi, Renaud Louis, Lars I. Andersson, Scott S. Wagers, Sven-Erik Dahlén, Kian Fan Chung, Ian M. Adcock, Martijn A. Spruit, *on behalf of the U-BIOPRED study group*

|  | **ACQ5** | **AQLQ** | **ESS** | **HADS-A** | **HADS-D** | **MARS** | **SNOT20** |
| --- | --- | --- | --- | --- | --- | --- | --- |
| **ACQ5** |  |  |  |  |  |  |  |
| **AQLQ** | -0.77***  (383) |  |  |  |  |  |  |
| **ESS** | 0.19***  (357) | -0.24***  (381) |  |  |  |  |  |
| **HADS-A** | 0.38***  (361) | -0.48***  (386) | 0.26***  (365) |  |  |  |  |
| **HADS-D** | 0.37***  (361) | -0.46***  (386) | 0.32***  (365) | 0.75***  (391) |  |  |  |
| **MARS** | -0.20***  (374) | 0.14**  (399) | -0.05  (373) | -0.11* (380) | -0.09  (380) |  |  |
| **SNOT20** | 0.44***  (326) | -0.50***  (342) | 0.34***  (327) | 0.51***  (328) | 0.50***  (328) | -0.15**  (335) |  |

**Table E1.** Interrelationship PROMS

| 0.00 - 0.19 | 0.20 - 0.39 | 0.40 - 0.59 | 0.60 - 0.79 | 0.80 - 1.00 |
| --- | --- | --- | --- | --- |
| very weak | weak | moderate | strong | very strong |

Spearman's rho was used to calculate correlations between variables. (n) represents the total number of sample values per analysis. * p<0.05; ** p<0.01; *** p<0.001
*Abbreviations:* ACQ5, Asthma Control Questionnaire; AQLQ, Asthma Quality of Life Questionnaire; ESS, Epworth Sleepiness Scale; HADS-A, Hospital Anxiety and Depression Scale, Anxiety subscale; HADS-D, Hospital Anxiety and Depression Scale, Depression subscale; MARS, Medication Adherence Report Scale; SNOT20, Sino-Nasal Outcomes Test.

**Table E2.** Baseline characteristics of patients who attended the longitudinal visit (n = 300) vs. excluded patients (n = 121)

|  | **Patients who attended longitudinal visit**  **(n=300)** | **Excluded patients**  **(n=121)** |
| --- | --- | --- |
| **Age, years** | 52.6 ± 13.2 | 50.3 ± 14.0 |
| **Age at diagnosis, years** | 26.0 (7.0 – 42.0) | 25.0 (10.0 – 43.0) |
| **Females, n (%)** | 179 (59.7) | 82 (67.8) |
| **BMI, kg/m^2^** | 29.0 ± 6.0 | 29.8 ± 7.0 |
| **BMI >30 kg/m^2^, n (%)** | 119 (39.7) | 45 (37.2) |
| **Smoking: current/former/never, n (%)** | 33 (11) /76 (25) /191 (64) | 9 (7)/ 39 (32)/ 73 (60) |
| **Serum IgE, IU/mL** | 130 (52 – 342) | 113 (45 – 374) |
| **Atopy test positive, n (%)** | 197 (65.7) | 78 (64.5) |
| **FEV_1_, %pred** | 66.2 ± 20.9 | 70.7 ± 22.0 |
| **FVC, %pred** | 87.5 ± 19.1 | 88.7 ± 19.9 |
| **FEV_1_/FVC ratio** | 0.62 ± 0.14 | 0.66 ± 0.14* |
| **FeNO, ppb** | 26 (15 – 47) | 25 (15 – 48) |
| **Blood neutrophils, cells/μL** | 4695 (3623 – 6760) | 5250 (4000 – 6995) |
| **Blood eosinophils, cells/μL** | 200 (100 – 400) | 225 (100 – 438) |
| **Asthma exacerbations <12 months** | 2 (1 – 3) | 2 (1 – 4) |

Summary variables are presented as n (%) for discrete variables, mean ± standard deviation for quantitative variables or median (Interquartile range) for skewed variables. * p<0.05, assessed with Independent Samples T-test
*Abbreviations*: BMI, body mass index; kg/m^2^, kilogram per square metre; IgE, Immunoglobuline E; IU/mL, international units per millilitre; FEV_1_, forced expiratory volume in the first second; %pred, percentage of the predicted value; FVC, forced vital capacity; FeNO, fractional exhaled nitric oxide; ppb, parts per billion; cells/μL, cells per microliter.

a) ACQ5 b) Number of asthma exacerbations <12 months at baseline c) AQLQ


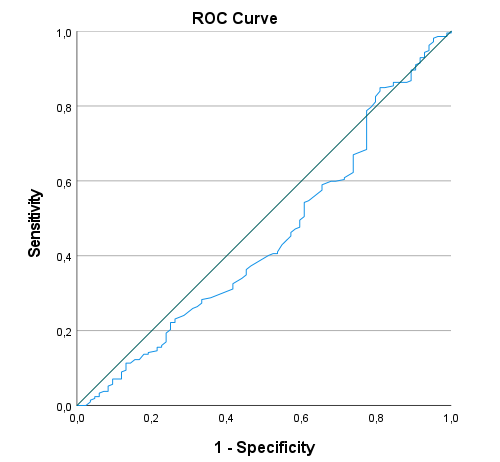

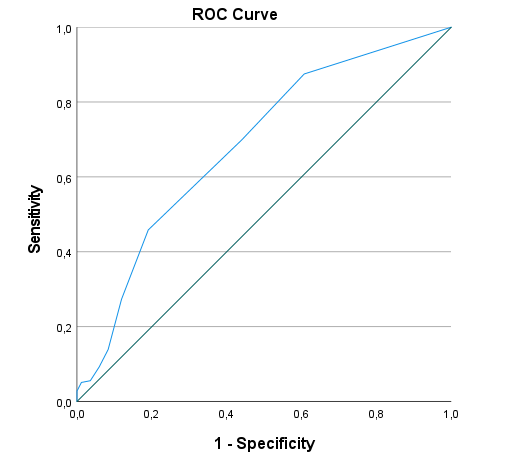

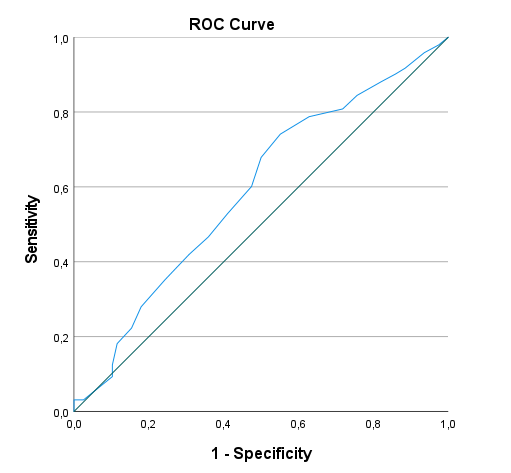


AUC: 0.684 ± 0.035

(95%CI 0.615 – 0.753; p<0.001)

AUC: 0.590 ± 0.039

(95%CI 0.514 – 0.666; p<0.05)

AUC: 0.448 ± 0.038

(95%CI 0.374 – 0.522; p=0.162


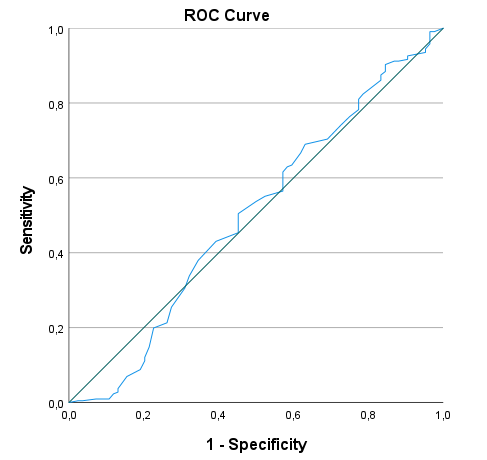

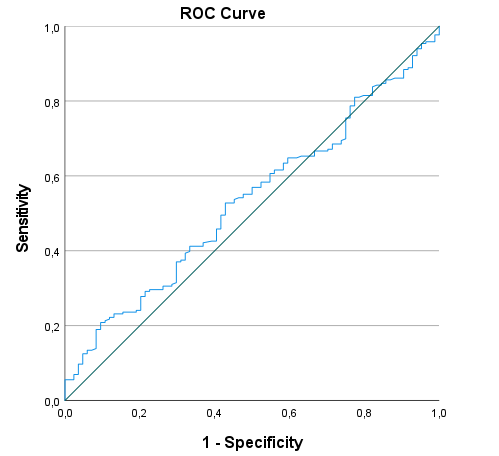
d) FEV1%pred e) Age f) BMI


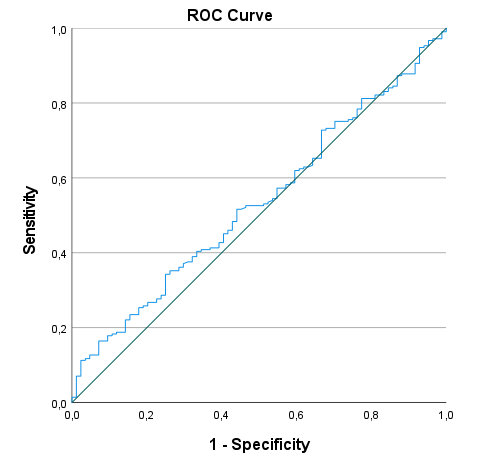


AUC: 0.533 ± 0.036

(95%CI 0.463 – 0.604; p=0.374

AUC: 0.533 ± 0.036

(95%CI 0.464 – 0.603; p=0.368)

AUC: 0.500 ± 0.039

(95%CI 0.424 – 0.577; p=0.993)

**Figure E1.** ROC (Receiver Operating Characteristics) analysis with AUC (Area Under The Curve) calculations for different PROMs and clinical variables in terms of predicting an asthma exacerbation.

*Abbreviations*: PROMs, patient-reported outcomes; ACQ5, Asthma Control Questionnaire; AQLQ, Asthma Quality of Life Questionnaire; FEV_1_, forced expiratory volume in the first second; BMI, body mass index.

**Acknowledgments (continued)**

The members of the U-BIOPRED Study Group are as follows: Abdel-Aziz MI, Department of Respiratory Medicine, Amsterdam UMC, University of Amsterdam, Amsterdam, The Netherlands; Adcock IM, National Heart and Lung Institute, Imperial College, London, UK; Andersson LI, Department of Respiratory Medicine, Karolinska University Hospital, Stockholm, Sweden; Auffray C, European Institute for Systems Biology and Medicine, CNRS-ENS-UCBL-INSERM, Lyon, France; Badi YE, National Heart and Lung Institute, Imperial College, London, UK; Bakke P , Department of Clinical Science, University of Bergen, Bergen, Norway; Bansal AT, Acclarogen Ltd, St. John’s Innovation Centre, Cambridge, UK; Baribaud F, Janssen R&D, LLC, Spring House, PA, USA; Bates S, Respiratory Therapeutic Unit, GSK, London, UK; Bel EH, Academic Medical Centre, University of Amsterdam, Amsterdam, The Netherlands; Bigler J, Previously Amgen Inc; Billing B, Department of Respiratory Medicine, Karolinska University Hospital, Stockholm, Sweden; Bisgaard H, COPSAC, Copenhagen Prospective Studies on Asthma in Childhood, Herlev and Gentofte Hospital, University of Copenhagen, Copenhagen, Denmark; Boedigheimer MJ, Amgen Inc.; Thousand Oaks, USA; Bønnelykke K, COPSAC, Copenhagen Prospective Studies on Asthma in Childhood, Herlev and Gentofte Hospital, University of Copenhagen, Copenhagen, Denmark; Brandsma J, University of Southampton, Southampton, UK; Brinkman P, Academic Medical Centre, University of Amsterdam, Amsterdam, The Netherlands; Bucchioni E , Chiesi Pharmaceuticals SPA, Parma, Italy; Burg D, Centre for Proteomic Research, Institute for Life Sciences, University of Southampton, Southampton, UK; Bush A, National Heart and Lung Institute, Imperial College, London, UK; Royal Brompton and Harefield NHS trust, UK; Caruso M, Dept. Biomedical and Biotechnological Sciences, University of Catania, Catania, Italy; Chalekis R, Institute of Environmental Medicine, Centre for Allergy Research, Karolinska Institutet, Stockholm, Sweden; Chanez P, Assistance publique des Hôpitaux de Marseille - Clinique des bronches, allergies et sommeil, Aix Marseille Université, Marseille, France ; Chung FK, National Heart and Lung Institute, Imperial College, London, UK; Checa T, Institute of Environmental Medicine, Centre for Allergy Research, Karolinska Institutet, Stockholm, Sweden; Compton CH, Respiratory Therapeutic Unit, GSK, London, UK; Corfield J, Areteva R&D, Nottingham, UK; Cunoosamy D, Sanofi, Cambridge, USA; D’Amico A, University of Rome ‘Tor Vergata’, Rome Italy; Dahlén B, Department of Respiratory Medicine, Karolinska University Hospital & Centre for Allergy Research, Karolinska Institutet, Stockholm, Sweden; Dahlén SE, Institute of Environmental Medicine, Centre for Allergy Research, Karolinska Institutet, and Department of Respiratory Medicine, Karolinska University Hospital, Stockholm, Sweden; De Meulder B, European Institute for Systems Biology and Medicine, CNRS-ENS-UCBL-INSERM, Lyon, France; Djukanovic R, NIHR Southampton Respiratory Biomedical Research Unit and Clinical and Experimental Sciences, Southampton, UK; Erpenbeck VJ, Translational Medicine, Respiratory Profiling, Novartis Institutes for Biomedical Research, Basel, Switzerland; Erzen D, Boehringer Ingelheim Pharma GmbH & Co. KG, Biberach, Germany; Fichtner K, Boehringer Ingelheim Pharma GmbH & Co. KG, Biberach, Germany; Fleming LJ, National Heart and Lung Institute, Imperial College, London, UK, Royal Brompton and Harefield NHS trust, UK; Formaggio E, Previously CROMSOURCE, Verona Italy; Fowler SJ, Division of infection, immunity and respiratory medicine, School of biological sciences, University of Manchester, Manchester University NHS Foundation Trust, Manchester Academic Health Science Centre, Manchester, United Kingdom; Frey U, University Children’s Hospital, Basel, Switzerland; Gahlemann M, Boehringer Ingelheim (Schweiz) GmbH, Basel, Switzerland; Geiser T, Department of Respiratory Medicine, University Hospital Bern, Switzerland; Goss V, NIHR Respiratory Biomedical Research Unit, University Hospital Southampton NHS Foundation Trust, Integrative Physiology and Critical Illness Group, Clinical and Experimental Sciences, Sir Henry Wellcome Laboratories, Faculty of Medicine, University of Southampton, Southampton, UK; Guo Y, Data Science Institute, Imperial College, London, UK; Hashimoto S, Academic Medical Centre, University of Amsterdam, Amsterdam, The Netherlands; Haughney J, International Primary Care Respiratory Group, Aberdeen, Scotland; Hedlin G, Dept. Women’s and Children’s Health & Centre for Allergy Research, Karolinska Institutet, Stockholm, Sweden; Hekking PW, Academic Medical Centre, University of Amsterdam, Amsterdam, The Netherlands; Higenbottam T, Allergy Therapeutics, West Sussex, UK; Hohlfeld JM, Fraunhofer Institute for Toxicology and Experimental Medicine, Hannover, Germany; Holweg C, Respiratory and Allergy Diseases, Genentech, San Francisco, USA; Horváth I, Department of Pulmonology and Department of Public Health, Semmelweis University, Budapest, Hungary; Howarth P, NIHR Southampton Respiratory Biomedical Research Unit, Clinical and Experimental Sciences and Human Development and Health, Southampton, UK; James AJ, Institute of Environmental Medicine, Centre for Allergy Research, Karolinska Institutet, Stockholm, Sweden; Knowles RG, Knowles Consulting Ltd, Stevenage. UK; Kolmert J, Institute of Environmental Medicine, Centre for Allergy Research, Karolinska Institutet, Stockholm, Sweden; Konradsen J, Dept. Women’s and Children’s Health & Centre for Allergy Research, Karolinska Institutet, Stockholm, Sweden; Krug N, Fraunhofer Institute for Toxicology and Experimental Medicine, Hannover, Germany; Lazarinis N, Department of Respiratory Medicine, Karolinska University Hospital & Centre for Allergy Research, Karolinska Institutet, Stockholm, Sweden; Li CX, Department of Medicine Solna, Karolinska Institutet, Stockholm, Sweden; Loza MJ, Janssen R&D, LLC, Spring House, PA, USA; Lutter R, Academic Medical Centre, University of Amsterdam, Amsterdam, The Netherlands; Manta A, Roche Diagnostics GmbH, Mannheim, Germany; Masefield S, European Lung Foundation, Sheffield, UK; Maitland-van der Zee Anke-Hilse, Department of Respiratory Medicine, Amsterdam UMC, University of Amsterdam, Amsterdam, The Netherlands; Matthews JG, Respiratory and Allergy Diseases, Genentech, San Francisco, USA; Mazein A, European Institute for Systems Biology and Medicine, CNRS-ENS-UCBL-INSERM, Lyon, France; Middelveld RJM, Centre for Allergy Research, Karolinska Institutet, Stockholm, Sweden; Miralpeix M, Almirall, Barcelona, Spain; Montuschi P, Università Cattolica del Sacro Cuore, Milan, Italy; Murray CS, Division of infection, immunity and respiratory medicine, School of biological sciences, University of Manchester, Manchester University NHS Foundation Trust, and Manchester Academic Health Science Centre, Manchester, United Kingdom; Musial J, Dept. of Medicine, Jagiellonian University Medical College, Krakow, Poland; Mumby S, National Heart and Lung Institute, Imperial College, London, UK; Myles D, Respiratory Therapeutic Unit, GSK, London, UK; Nordlund B, Dept. Women’s and Children’s Health & Centre for Allergy Research, Karolinska Institutet, Stockholm, Sweden; Pandis I, Data Science Institute, Imperial College, London, UK; Pavlidis S, National Heart and Lung Institute, Imperial College, London, UK; Postle A, University of Southampton, UK; Powel P, European Lung Foundation, Sheffield, UK; Praticò G, CROMSOURCE, Verona, Italy; Puig Valls M, CROMSOURCE, Barcelona, Spain; Rao N, Janssen R&D, LLC, Spring House, PA, USA; Reinke S, Institute of Environmental Medicine, Karolinska Institutet, Stockoholm, Sweden; Riley J, Respiratory Therapeutic Unit, GSK, London, UK; Roberts A, Asthma UK, London, UK; Roberts G, NIHR Southampton Respiratory Biomedical Research Unit, Clinical and Experimental Sciences and Human Development and Health, Southampton, UK; Rowe A, Janssen R&D, UK; Sandström T, Dept of Public Health and Clinical Medicine, Umeå University, Umeå, Sweden; Schofield JPR, Centre for Proteomic Research, Institute for Life Sciences, University of Southampton, Southampton, UK; Seibold W, Boehringer Ingelheim Pharma GmbH, Biberach, Germany; Shaw DE, Respiratory Research Unit, University of Nottingham, UK; Sigmund R, Boehringer Ingelheim Pharma GmbH & Co. KG, Biberach, Germany; Singer F, Pediatric Respiratory Medicine, Department of Pediatrics, Inselspital, Bern University Hospital, University of Bern, Bern, Switzerland; Skipp PJ, Centre for Proteomic Research, Institute for Life Sciences, University of Southampton, Southampton, UK; Smicker M, Sanofi, Cambridge, USA; Sousa AR, Respiratory Therapeutic Unit, GSK, London, UK; Sparreman-Mikus M, Department of Medicine Huddinge, Karolinska Institutet, and Department of Respiratory Medicine, Karolinska University Hospital, Stockholm, Sweden; Sterk PJ, Academic Medical Centre, University of Amsterdam, Amsterdam, The Netherlands; Ström M, Department of Medicine Solna, Karolinska Institutet, Stockholm, Sweden; Sun K, Data Science Institute, Imperial College, London, UK; Thornton B, MSD, USA; Uddin M, AstraZeneca BioPharmaceuticals R&D, Gothenburg, Sweden; Versi A, National Heart and Lung Institute, Imperial College, London, UK; Vestbo J, Centre for Respiratory Medicine and Allergy, Institute of Inflammation and Repair, University of Manchester and University Hospital of South Manchester, Manchester Academic Health Sciences Centre, Manchester, United Kingdom; Vissing NH, COPSAC, Copenhagen Prospective Studies on Asthma in Childhood, Herlev and Gentofte Hospital, University of Copenhagen, Copenhagen, Denmark; Wagers SS, BioSci Consulting, Maasmechelen, Belgium; Wheelock AM, Respiratory Medicine Unit, Department of Medicine Solna and Center for Molecular Medicine, Karolinska Institutet, Stockholm, Sweden and Department of Respiratory Medicine and Allergy, Karolinska University Hospital Solna, Stockholm, Sweden; Wheelock CE, Institute of Environmental Medicine, Centre for Allergy Research, Karolinska Institutet, Stockholm, Sweden; Wilson SJ, Histochemistry Research Unit, Faculty of Medicine, University of Southampton, Southampton, UK; Yasinska V, Department of Medicine Huddinge, Karolinska Institutet, and Department of Respiratory Medicine, Karolinska University Hospital, Stockholm, Sweden; Zounemat Kermani N, Data Science Institute, Imperial College, London, UK.

The U-BIOPRED consortium wishes to acknowledge the help and expertise of the following individuals and groups without whom, the study would not have been possible.

Investigators and contributors: Ahmed H, European Institute for Systems Biology and Medicine, CNRS-ENS-UCBL-INSERM, Lyon, France; Aliprantis Antonios, Merck Research Laboratories, Boston, USA; Allen David, North West Severe Asthma Network, Pennine Acute Hospital NHS Trust, UK; Alving Kjell, Dept Women’s & Children’s Health, Uppsala University, Uppsala, Sweden; Badorrek P, Fraunhofer ITEM; Hannover, Germany; Balgoma David, Centre for Allergy Research, Karolinska Institutet, Stockholm, Sweden; Ballereau S, European institute for Systems Biology and Medicine, University of Lyon, France; Barber Clair, NIHR Southampton Respiratory Biomedical Research Unit and Clinical and Experimental Sciences, Southampton, UK; Batuwitage Manohara Kanangana, Data Science Institute, Imperial College, London, UK; Bautmans An, MSD, Brussels, Belgium; Bedding A, Roche Diagnostics GmbH, Mannheim, Germany; Behndig AF, Umeå University, Umea, Sweden; Beleta Jorge, Almirall S.A., Barcelona, Spain; Berglind A, MSD, Brussels, Belgium; Bochenek Grazyna, II Department of Internal Medicine, Jagiellonian University Medical College, Krakow, Poland; Braun Armin, Fraunhofer Institute for Toxicology and Experimental Medicine, Hannover, Germany; Campagna D, Department of Clinical and Experimental Medicine, University of Catania, Catania, Italy; Carayannopoulos Leon, Previously at: MSD, USA; Casaulta C, University Children’s Hospital of Bern, Switzerland; Chaiboonchoe A, European Institute for Systems Biology and Medicine, CNRS-ENS-UCBL-INSERM, Lyon, France; Chaleckis Romanas, Centre of Allergy Research, Karolinska Institutet, Stockholm, Sweden; Davison Timothy Janssen R&D, LLC, Spring House, PA, USA; De Alba Jorge, Almirall S.A., Barcelona, Spain; De Lepeleire Inge, MSD, Brussels, BE; Dekker Tamara, Academic Medical Centre, University of Amsterdam, Amsterdam, The Netherlands; Delin Ingrid, Centre for Allergy Research, Karolinska Institutet, Stockholm, Sweden; Dennison P, NIHR Southampton Respiratory Biomedical Research Unit, Clinical and Experimental Sciences, NIHR-Wellcome Trust Clinical Research Facility, Faculty of Medicine, University of Southampton, Southampton, UK; Dijkhuis Annemiek, Academic Medical Centre, University of Amsterdam, Amsterdam, The Netherlands; Dodson Paul, AstraZeneca BioPharmaceuticals R&D, Gothenburg, Sweden; Draper Aleksandra, BioSci Consulting, Maasmechelen, Belgium; Dyson K, CROMSOURCE; Stirling, UK; Edwards Jessica, Asthma UK, London, UK; El Hadjam L, European Institute for Systems Biology and Medicine, University of Lyon; Emma Rosalia, Department of Biomedical and Biotechnological Sciences, University of Catania, Catania, Italy; Ericsson Magnus, Karolinska University Hospital, Stockholm, Sweden; Faulenbach C, Fraunhofer ITEM; Hannover, Germany; Flood Breda, European Federation of Allergy and Airways Diseases Patient’s Associations, Brussels, Belgium; Galffy G, Semmelweis University, Budapest, Hungary; Gallart Hector, Centre for Allergy Research, Karolinska Institutet, Stockholm, Sweden; Garissi D, Global Head Clinical Research Division, CROMSOURCE, Italy; Gent J, Royal Brompton and Harefield NHS Foundation Trust, London, UK; Gerhardsson de Verdier M, AstraZeneca BioPharmaceuticals R&D, Gothenburg, Sweden; Gibeon D, National Heart and Lung Institute, Imperial College, London, UK; Gomez Cristina, Centre for Allergy Research, Karolinska Institutet, Stockholm, Sweden; Gove Kerry, NIHR Southampton Respiratory Biomedical Research Unit and Clinical and Experimental Sciences, Southampton, UK; Gozzard Neil, UCB, Slough, UK; Guillmant-Farry E, Royal Brompton Hospital, London, UK; Henriksson E, Karolinska University Hospital & Karolinska Institutet, Stockholm, Sweden; Hewitt Lorraine, NIHR Southampton Respiratory Biomedical Research Unit, Southampton, UK; Hoda U, Imperial College, London, UK; Hu Richard, Amgen Inc. Thousand Oaks, USA; Hu Sile, National Heart and Lung Institute, Imperial College, London, UK; Hu X, Amgen Inc.; Thousand Oaks, USA; Jeyasingham E, UK Clinical Operations, GSK, Stockley Park, UK; Johnson K, Centre for respiratory medicine and allergy, Institute of Inflammation and repair, University Hospital of South Manchester, NHS Foundation Trust, Manchester, UK; Jullian N, European Institute for Systems Biology and Medicine, University of Lyon; Kamphuis Juliette, Longfonds, Amersfoort, The Netherlands; Kennington Erika J., Asthma UK, London, UK; Kerry Dyson, CromSource, Stirling, UK; Kerry G, Centre for respiratory medicine and allergy, Institute of Inflammation and repair, University Hospital of South Manchester, NHS Foundation Trust, Manchester, UK; Klüglich M, Boehringer Ingelheim Pharma GmbH & Co. KG, Biberach, Germany; Knobel Hugo, Philips Research Laboratories, Eindhoven, The Netherlands; Knox Alan J, Respiratory Research Unit, University of Nottingham, Nottingham, UK; Kolmert Johan, Centre for Allergy Research, Karolinska Institutet, Stockholm, Sweden; Konradsen J R, Dept. Women’s and Children’s Health & Centre for Allergy Research, Karolinska Institutet, Stockholm, Sweden; Kots Maxim, Chiesi Pharmaceuticals, SPA, Parma, Italy; Kretsos Kosmas, UCB, Slough, UK; Krueger L, University Children's Hospital Bern, Switzerland; Kuo Scott, National Heart and Lung Institute, Imperial College, London, UK; Kupczyk Maciej, Centre for Allergy Research, Karolinska Institutet, Stockholm, Sweden; Lambrecht Bart, University of Gent, Gent, Belgium; Lantz A-S, Karolinska University Hospital & Centre for Allergy Research, Karolinska Institutet, Stockholm, Sweden; Larminie Christopher, GSK, London, UK; Larsson L X, AstraZeneca BioPharmaceuticals R&D, Gothenburg, Sweden; Latzin P, University Children’s Hospital of Bern, Bern, Switzerland; Lazarinis N, Karolinska University Hospital & Karolinska Institutet, Stockholm, Sweden; Lefaudeux Diane, European Institute for Systems Biology and Medicine, CNRS-ENS-UCBL-INSERM, Lyon, France; Lemonnier N, European Institute for Systems Biology and Medicine, CNRS-ENS-UCBL-INSERM, Lyon, France; Li Chuan-Xing, Respiratory Medicine Unit, Department of Medicine Solna and Center for Molecular Medicine, Karolinska Institutet, Stockholm, Sweden; and Department of Respiratory Medicine and Allergy, Karolinska University Hospital Solna, Stockholm, Sweden; Lone-Latif Saeeda, Academic Medical Centre, University of Amsterdam, Amsterdam, The Netherlands; Lowe L A, Centre for respiratory medicine and allergy, Institute of Inflammation and repair, University Hospital of South Manchester, NHS Foundation Trust, Manchester, UK; Manta Alexander, Roche Diagnostics GmbH, Mannheim, Germany; Marouzet Lisa, NIHR Southampton Respiratory Biomedical Research Unit, Southampton, UK; Martin Jane, NIHR Southampton Respiratory Biomedical Research Unit, Southampton, UK; Mathon Caroline, Centre of Allergy Research, Karolinska Institutet, Stockholm, Sweden; McEvoy L, University Hospital, Department of Pulmonary Medicine, Bern, Switzerland; Meah Sally, National Heart and Lung Institute, Imperial College, London, UK; Menzies-Gow A, Royal Brompton and Harefield NHS Foundation Trust, London, UK; Metcalf Leanne, Previously at: Asthma UK, London, UK; Meiser Andrea, Data Science Institute, Imperial College, London, UK; Mikus Maria, Science for Life Laboratory & The Royal Institute of Technology, Stockholm, Sweden; Monk Philip, Synairgen Research Ltd, Southampton, UK; Mores N, Università Cattolica del Sacro Cuore, Milan, Italy; Naz Shama, Centre for Allergy Research, Karolinska Institutet, Stockholm, Sweden; Nething K, Boehringer Ingelheim Pharma GmbH & Co. KG; Biberach, Germany; Nicholas Ben, University of Southampton, Southampton, UK; Nihlén U, Previously AstraZeneca BioPharmaceuticals R&D, Gothenburg, Sweden; Nilsson Peter, Science for Life Laboratory & The Royal Institute of Technology, Stockholm, Sweden; Niven R, North West Severe Asthma Network, University Hospital South Manchester, UK; Nordlund B, Dept. Women’s and Children’s Health & Centre for Allergy Research, Karolinska Institutet, Stockholm, Sweden; Nsubuga S, Royal Brompton Hospital, London, UK Pacino Antonio, Lega Italiano Anti Fumo, Catania, Italy; Palkonen Susanna, European Federation of Allergy and Airways Diseases Patient’s Associations, Brussels, Belgium; Pahus L, Assistance publique des Hôpitaux de Marseille, Clinique des bronches, allergies et sommeil Espace Éthique Méditerranéen, Aix-Marseille Université, Marseille, France ; Pellet J, European Institute for Systems Biology and Medicine, CNRS-ENS-UCBL-INSERM, Lyon, France; Pennazza Giorgio, Unit of Electronics for Sensor Systems, Department of Engineering, Campus Bio-Medico University of Rome, Rome, Italy; Petrén Anne, Centre for Allergy Research, Karolinska Institutet, Stockholm, Sweden; Pink Sandy, NIHR Southampton Respiratory Biomedical Research Unit, Southampton, UK; Pison C, European Institute for Systems Biology and Medicine, CNRS-ENS-UCBL-INSERM, Lyon, France; Rahman-Amin Malayka, Previously at: Asthma UK, London, UK; Ravanetti Lara, Academic Medical Centre, University of Amsterdam, Amsterdam, The Netherlands; Ray Emma, NIHR Southampton Respiratory Biomedical Research Unit, Southampton, UK; Reinke Stacey, Centre for Allergy Research, Karolinska Institutet, Stockholm, Sweden; Reynolds Leanne, Previously at: Asthma UK, London, UK; Riemann K, Boehringer Ingelheim Pharma GmbH & Co. KG; Biberach, Germany; Robberechts Martine, MSD, Brussels, Belgium; Rocha J P, Royal Brompton and Harefield NHS Foundation Trust; Rossios C, National Heart and Lung Institute, Imperial College, London, UK; Russell Kirsty, National Heart and Lung Institute, Imperial College, London, UK; Rutgers Michael, Longfonds, Amersfoort, The Netherlands; Santini G, Università Cattolica del Sacro Cuore, Milan, Italy; Santonico Marco, Unit of Electronics for Sensor Systems, Department of Engineering, Campus Bio-Medico University of Rome, Rome, Italy; Saqi M, European Institute for Systems Biology and Medicine, CNRS-ENS-UCBL-INSERM, Lyon, France; Schoelch Corinna, Boehringer Ingelheim Pharma GmbH & Co. KG, Biberach, Germany; Scott S, North West Severe Asthma Network, Countess of Chester Hospital, UK; Sehgal N, North West Severe Asthma Network; Pennine Acute Hospital NHS Trust; Selby A, NIHR Southampton Respiratory Biomedical Research Unit, Clinical and Experimental Sciences and Human Development and Health, Southampton, UK; Sjödin Marcus, Centre for Allergy Research, Karolinska Institutet, Stockholm, Sweden; Smids Barbara, Academic Medical Centre, University of Amsterdam, Amsterdam, The Netherlands; Smith Caroline, NIHR Southampton Respiratory Biomedical Research Unit, Southampton, UK; Smith Jessica, Asthma UK, London, UK; Smith Katherine M., University of Nottingham, UK; Söderman P, Dept. Women’s and Children’s Health, Karolinska Institutet, Stockholm, Sweden; Sogbesan A, Royal Brompton and Harefield NHS Foundation Trust, London, UK; Spycher F, University Hospital Department of Pulmonary Medicine, Bern, Switzerland; Staykova Doroteya, University of Southampton, Southampton, UK; Stephan S, Centre for respiratory medicine and allergy, Institute of Inflammation and repair, University Hospital of South Manchester, NHS Foundation Trust, Manchester, UK; Stokholm J, University of Copenhagen and Danish Pediatric Asthma Centre Denmark; Strandberg K, Karolinska University Hospital & Karolinska Institutet, Stockholm, Sweden; Sunther M, Centre for respiratory medicine and allergy, Institute of Inflammation and repair, University Hospital of South Manchester, NHS Foundation Trust, Manchester, UK; Szentkereszty M, Semmelweis University, Budapest, Hungary; Tamasi L, Semmelweis University, Budapest, Hungary; Tariq K, NIHR Southampton Respiratory Biomedical Research Unit, Clinical and Experimental Sciences, NIHR-Wellcome Trust Clinical Research Facility, Faculty of Medicine, University of Southampton, Southampton, UK; Thörngren John-Olof, Karolinska University Hospital, Stockholm, Sweden; Thorsen Jonathan, COPSAC, Copenhagen Prospective Studies on Asthma in Childhood, Herlev and Gentofte Hospital, University of Copenhagen, Copenhagen, Denmark; Valente S, Università Cattolica del Sacro Cuore, Milan, Italy; van Aalderen W M, Academic Medical Centre, University of Amsterdam, Amsterdam, The Netherlands; van de Pol Marianne, Academic Medical Centre, University of Amsterdam, Amsterdam ,The Netherlands; van Drunen C M, Academic Medical Centre, University of Amsterdam, Amsterdam, The Netherlands; Van Eyll Jonathan, UCB, Slough, UK; Versnel Jenny, Previously at: Asthma UK, London, UK; Vink Anton, Philips Research Laboratories, Eindhoven, The Netherlands; von Garnier C, University Hospital Bern, Switzerland; Vyas A, North west Severe Asthma Network, Lancashire Teaching Hospitals NHS Trust, UK; Wagener A H, Academic Medical Center Amsterdam, Amsterdam, The Netherlands; Wald Frans, Boehringer Ingelheim Pharma GmbH & Co. KG, Biberach, Germany; Walker Samantha, Asthma UK, London, UK; Ward Jonathan, Histochemistry Research Unit, Faculty of Medicine, University of Southampton, Southampton, UK; Wetzel Kristiane, Boehringer Ingelheim Pharma GmbH, Biberach, Germany; Wiegman Coen, National Heart and Lung Institute, Imperial College, London, UK; Weiszhart Z, Semmelweis University, Budapest, Hungary; Williams Siân, International Primary Care Respiratory Group, Aberdeen, Scotland; Yang Xian, Data Science Institute, Imperial College, London, UK; Yeyasingham Elizabeth, UK Clinical Operations, GSK, Stockley Park, UK; Yu W, Amgen Inc.; Thousand Oaks, USA; Zetterquist W, Dept. Women’s and Children’s Health & Centre for Allergy Research, Karolinska Institutet, Stockholm, Sweden; Zolkipli Z, NIHR Southampton Respiratory Biomedical Research Unit, Clinical and Experimental Sciences and Human Development and Health, Southampton, UK; Zwinderman A H, Academic Medical Centre, University of Amsterdam, The Netherlands.

Partner organisations: Novartis Pharma AG; University of Southampton, Southampton, UK; Academic Medical Centre, University of Amsterdam, Amsterdam, The Netherlands; Imperial College London, London, UK; University of Catania, Catania, Italy; University of Rome ‘Tor Vergata’, Rome, Italy; Hvidore Hospital, Hvidore, Denmark; Jagiellonian Univ. Medi.College, Krakow, Poland; University Hospital, Inselspital, Bern, Switzerland; Semmelweis University, Budapest, Hungary; University of Manchester, Manchester, UK; Université d'Aix-Marseille, Marseille, France; Fraunhofer Institute, Hannover, Germany; University Hospital, Umea, Sweden; Ghent University, Ghent, Belgium; Ctr. Nat. Recherche Scientifique, Villejuif, France; Università Cattolica del Sacro Cuore, Rome, Italy; University Hospital, Copenhagen, Denmark; Karolinska Institutet, Stockholm, Sweden; Nottingham University Hospital, Nottingham, UK; University of Bergen, Bergen, Norway; Netherlands Asthma Foundation, Leusden, NL; European Lung Foundation, Sheffield, UK; Asthma UK, London, UK; European Fed. of Allergy and Airways Diseases Patients’ Associations, Brussels, Belgium; Lega Italiano Anti Fumo, Catania, Italy; International Primary Care Respiratory Group, Aberdeen, Scotland; Philips Research Laboratories, Eindhoven, NL; Synairgen Research Ltd, Southampton, UK; Aerocrine AB, Stockholm, Sweden; BioSci Consulting, Maasmechelen, Belgium; Almirall; AstraZeneca; Boehringer Ingelheim; Chiesi; GlaxoSmithKline; Roche; UCB; Janssen Biologics BV; Amgen NV; Merck Sharp & Dohme Corp.

Members of the ethics board: Jan-Bas Prins, biomedical research, LUMC, the Netherlands; Martina Gahlemann, clinical care, BI, Germany; Luigi Visintin, legal affairs, LIAF, Italy; Hazel Evans, paediatric care, Southampton, UK; Martine Puhl, patient representation (co-chair), NAF, the Netherlands; Lina Buzermaniene, patient representation, EFA, Lithuania; Val Hudson, patient representation, Asthma UK; Laura Bond, patient representation, Asthma UK; Pim de Boer, patient representation and pathobiology, IND; Guy Widdershoven, research ethics, VUMC, the Netherlands; Ralf Sigmund, research methodology and biostatistics, BI, Germany.

The patient input platform: Amanda Roberts, UK; David Supple (chair), UK; Dominique Hamerlijnck, The Netherlands; Jenny Negus, UK; Juliёtte Kamphuis, The Netherlands; Lehanne Sergison, UK; Luigi Visintin, Italy; Pim de Boer (co-chair), The Netherlands; Susanne Onstein, The Netherlands.

Members of the safety monitoring board: William MacNee, clinical care; Renato Bernardini, clinical pharmacology; Louis Bont, paediatric care and infectious diseases; Per-Ake Wecksell, patient representation; Pim de Boer, patient representation and pathobiology (chair); Martina Gahlemann, patient safety advice and clinical care (co-chair); Ralf Sigmund, bio-informatician.
